# Decoding the inflammatory-osteogenic axis in ankylosing spondylitis: mechanisms, dysregulation, and emerging therapeutic interventions

**DOI:** 10.3389/fimmu.2025.1633318

**Published:** 2025-09-12

**Authors:** Afroza Parvin, Ashish Ranjan Sharma, Md. Ashraful Hasan, Garima Sharma, Mohammad Mahfuz Ali Khan Shawan, Eun Min Seo, Md. Mahmudul Hasan, Sang-Soo Lee

**Affiliations:** ^1^ Department of Biochemistry and Molecular Biology, Jahangirnagar University, Dhaka, Bangladesh; ^2^ Department of Pharmacology and Therapeutics, University of Manitoba, Winnipeg, MB, Canada; ^3^ Institute for Skeletal Aging and Orthopedic Surgery, Hallym University-Chuncheon Sacred Heart Hospital, Chuncheon-si, Gangwon-Do, Republic of Korea; ^4^ Department of Biomedical Science and Institute of Bioscience and Biotechnology, Kangwon National University, Chuncheon, Gangwon-Do, Republic of Korea

**Keywords:** ankylosing spondylitis, HLA-B27, PGISp mouse, ERAP1 gene, therapeutic targets

## Abstract

Ankylosing spondylitis (AS) is a chronic autoimmune disorder that primarily affects young people. Although genetic and environmental factors have been implicated in the pathogenesis of AS, the etiology of this condition remains unclear. Observations indicate that individuals possessing the human leukocyte antigen (HLA)-B27 allele exhibit elevated risk factors, as any mutation within this gene could potentially result in the development of AS in the future. However, it is interesting to note that many AS patients do not carry this gene, inferring the involvement of other genetic and nongenetic factors in the development of the disease. As the exact mechanisms remain unknown, no target-specific treatments exist to cure AS. Nonetheless, some treatment regimens have been devised to alleviate AS symptoms. This review thoroughly examines the molecular mechanisms implicated in AS, encompassing insights into the significance of pivotal biomarkers, such as extracellular matrix metabolites, immune cell dynamics, gut microbiota interactions, the Wnt signaling pathway, and its inhibitors. Furthermore, a thorough evaluation of the different mouse models used in AS research has been reviewed, which is crucial for understanding disease pathways and assessing treatment methods. In addition, significant progress in developing effective treatment strategies for AS, along with drugs available for treatment and ongoing clinical trials, has been summarized. A comprehensive understanding of experimental mouse models, along with insights into molecular mechanisms and biomarkers for AS, could aid researchers and physicians in discovering new treatment strategies for this challenging condition.

## 1 Introduction

A group of inflammatory rheumatological conditions known as spondyloarthritis (SpA) impacts the spine, joints, and other organ systems. Axial spondyloarthritis (axSpA) and peripheral spondyloarthritis are the two primary categories into which SpA is often divided. The axial skeleton, which includes the spine, chest, and sacroiliac joints—the joints that connect the sacrum to the pelvis—is the main target of axial spondyloarthritis (1). On the other hand, peripheral spondyloarthritis primarily affects the knees, fingers, and toes, causing discomfort, stiffness, and swelling in these joints (2). Chronic inflammatory back pain and structural alterations to the sacroiliac joints are hallmarks of ankylosing spondylitis (AS), a more severe or advanced form of axial spondyloarthritis (3). Traditionally, X-ray imaging was used extensively for diagnosis since it could identify sacroiliitis, an inflammation of the sacroiliac joints. Two subcategories have been established to describe the progression of the disease using this imaging-based method: radiographic axial spondyloarthritis (r-axSpA) and non-radiographic axial spondyloarthritis (nr-axSpA). The r-axSpA subtype is often used interchangeably with ankylosing spondylitis (AS) and is characterized by structural changes that are visible on X-rays. People who exhibit the clinical and symptomatic characteristics of axSpA but do not exhibit any discernible alterations on radiographic imaging are said to have non-radiographic axial spondyloarthritis (nr-axSpA) (4). Early identification and tailored treatment plans for individuals with SpA require an understanding of the differences between r-axSpA and nr-axSpA.

The Assessment of SpondyloArthritis International Society (ASAS) criteria for axSpA encompass both non-radiographic and radiographic forms, hence possessing the capability to supplement the modified New York criteria for AS (5–10). The ASAS categorizes patients into two groups based on imaging characteristics (11). In contrast to non-radiographic axial spondyloarthritis (nr-axSpA), AS adheres to the modified New York criteria, including radiographic sacroiliitis assessment (12). Each axSpA subtype exhibits axial, peripheral, and extra-articular manifestations. Back pain, peripheral arthritis, and enthesitis manifest equally throughout the axSpA subtypes; however, uveitis is more prevalent in AS (13). In nr-axSpA patients, 4.9–11.6% develop radiographic sacroiliitis within two years (14, 15), and 19% do so after 10 years of follow-up (16). The hallmark symptom of axSpA is chronic inflammatory low back pain, which typically improves with physical activity and is often accompanied by morning stiffness. Musculoskeletal features may include arthritis, enthesitis, and dactylitis. Interestingly, reports of peripheral manifestations, particularly enthesitis and arthritis, are more prevalent in Latin America compared to Europe or the United States (17, 18).

AxSpA most commonly affects men in early adulthood, though both sexes can be impacted (1, 2). The disease involves complex immunological and biomechanical factors. For instance, intestinal inflammation and mechano-inflammatory stimuli contribute to bone marrow involvement, which is closely linked to enthesitis—inflammation at the sites where tendons or ligaments attach to bone (3). Several immune and stromal cell types are implicated in these inflammatory pathways, including mesenchymal stem cells, innate lymphoid cells, and γδ-T cells. Key molecular mediators include toll-like receptors and pro-inflammatory cytokines such as tumor necrosis factor (TNF), interleukin (IL)-17A, IL-22, IL-23, granulocyte-macrophage colony-stimulating factor (GM-CSF), and transforming growth factor-beta (TGF-β) (3). These immune interactions play a critical role in driving the pathogenesis and chronicity of axSpA.

Inflammation and osteoproliferation in the axial skeleton are key pathogenic events contributing to the clinical burden of AS (2). Human leukocyte antigen-B27 (HLA-B27) genetic variables are responsible for a significant amount of the estimated over 90% heritability of AS (6). Patients with AS may also have extra-articular symptoms such as psoriasis, uveitis, and ulcerative colitis in addition to axial and peripheral joint involvement (3). Notably, higher disease activity has been associated with the presence of HLA-B27 (4). The pathogenic role of intestinal inflammation in AS has been investigated using HLA-B27 transgenic (HLA-B27-Tg) rat models, which express human β2-microglobulin (hβ2m) and HLA-B27. These models exhibit intestinal inflammation resembling features seen in human AS. One proposed mechanism involves endoplasmic reticulum (ER) stress, which triggers the production of interleukin-23 (IL-23), a cytokine implicated in SpA pathogenesis. Interestingly, deletion of CHOP (C/EBP homologous protein; Ddit3^−/−^)—a transcription factor involved in ER stress signaling—was shown to affect this inflammatory pathway. In HLA-B27-Tg rats lacking CHOP, intestinal inflammation was not alleviated, but rather exacerbated, suggesting that CHOP may play a protective role against HLA-B27-induced gut inflammation (6). This finding indicates that CHOP may serve as a modulator of ER stress-mediated inflammatory responses in murine models of AS.

In North America and Europe, estimates of the prevalence of AS in adult populations typically fall between 0.20 and 0.25 percent. However, prevalence rates are greater in some populations, such as 0.29% among mainland Chinese military personnel and 0.35% among Northern Arctic tribes, which have the highest prevalence of HLA-B27 worldwide (19–21). In contrast, indigenous people in southern Africa, where HLA-B27 is essentially nonexistent, have extremely low rates of AS (19, 20). The prevalence of AS and HLA-B27 is strongly correlated across populations, according to these findings. The prevalence of HLA-B27 is likewise favorably correlated with the mean yearly incidence of AS (19, 22). However, variations in prevalence and incidence estimates across studies may be influenced by differences in methodological design, geographic region, case definitions, and demographic characteristics of the populations studied (18, 23). Further epidemiological insight is provided by data from Mexico, where patients with r-axSpA were predominantly male, had a higher prevalence of HLA-B27, presented at a younger age, and exhibited greater axial enthesopathy and disease activity than those with nr-axSpA. Additionally, r-axSpA patients had a higher incidence of uveitis and fewer cases of dactylitis (24). The HLA-B27 allele is absent in a significant percentage of patients with axSpA, including 40% of women and 37.5% of black patients (25–27). In addition, nr‐axSpA, a subtype of axSpA with a lower incidence of HLA-B27, is diagnosed more often in women (28–30). About 25% of patients with axSpA are HLA-B27 negative, according to pooled data from a Canadian cohort and three other local, national, and worldwide cohorts (1, 31). Up to 40% of patients with nr-axSpA may be HLA-B27 negative, according to other investigations (32). Furthermore, only around 20% of the total heredity of axSpA is thought to be due to HLA-B27 (33–36).

AS is a highly heritable autoimmune disease (18, 23, 24, 37, 38). The transition from inflammation to bone production in AS is one of its most debilitating characteristics. An unidentified process initiates inflammation at the junctions of tendons and ligaments with bone, resulting in enthesitis, followed by the development of bony projections (syndesmophytes) that fuse and cause ankylosis (39). As a result of the release of pro-inflammatory cytokines during the inflammatory phase, joint damage is caused by osteoclast activity, similar to other inflammatory arthropathies, such as rheumatoid arthritis (RA) (5). Patients with AS are susceptible to pathological fractures resulting from osteopenia or osteoporosis in both the axial and peripheral skeletons (40).

Numerous studies have sought to elucidate the relationship between inflammation and osteoproliferation, explicitly investigating whether inflammation directly induces osteoproliferation, subsides before bone formation, or whether the inflammatory and osteoproliferative phases are completely distinct (41). It has been observed that multiple mechanisms, including intramembranous, endochondral, and chondroidal ossification, may mediate osteoproliferation in AS (42, 43). The sites of initial inflammation are the intervertebral discs (IVDs) or sites where the annulus fibrosus (AF), outer fibers of the spinal, and sacroiliac ligaments attach. This inflammation leads to osteoproliferation, vertebral squaring, and the development of syndesmophytes at the vertebral corners, potentially leading to bridging and ankylosis. However, the cause of the initiation of inflammation and its development into bone growth and ankyloses remains unknown (44). Thus, in this article, we have tried to understand and discuss various molecular mechanisms of AS and updated the list of promising therapeutic targets and therapeutics for treating AS.

## 2 Known mechanisms of AS disease progression

Two distinguishing features of AS include syndesmophyte development and inflammation. Tseng et al. (2016) shed light on these issues by thoroughly examining illness progression in the proteoglycan-induced spondylitis (PGISp) mouse model of AS. Their findings indicated that osteoproliferation was exclusively observed at locations with prior inflammation, suggesting that inflammation and intervertebral disc degradation are critical for disease advancement and excessive bone formation (45). Tseng et al. discovered via histological investigation that inflammatory infiltration began at the periphery of the AF. Although AF was previously associated with inflammation in this model, it was not recognized as the beginning of inflammation in the spine (44, 46). Additionally, spinal enthesitis in the form of pannus development outside the AF has been observed in HLA-B27 transgenic rats (47) and SKG mice that have received curdlan or mannan treatment (48). They also demonstrated the expansion of ectopic chondroproliferation and inflammatory infiltration along the cortical bone surfaces of the nearby vertebrae. Although they do not offer conclusive evidence of enthesitis in these regions, it is common knowledge that longitudinal spinal ligaments frequently attach here (49, 50).

The necessity of inflammation for osteoproliferation in AS was underscored by the minimal effect of anti-tumor necrosis factor (anti-TNF) therapies on radiographic progression. Tseng et al. conducted a comprehensive histological examination revealing that AS’s early, intermediate, and late stages were marked by inflammation, hyperplasia, and ectopic chondrocyte formation. They demonstrated that early mesenchymal development commenced prior to resolution of inflammation (45).

**Figure 1:**
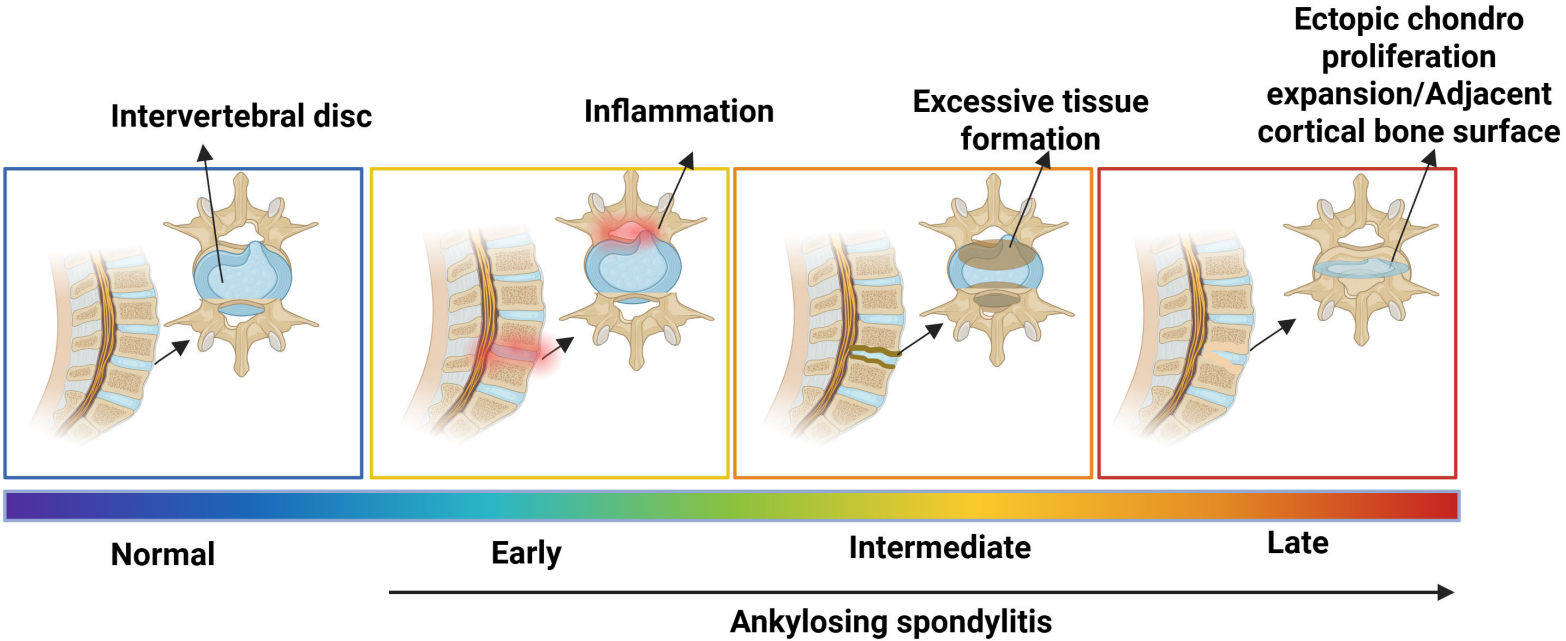
Recently identified biomarkers of nr-axSpA and AS. Inflammation to bone formation is a slow, sequential, but not parallel process. When there is IVD destruction, inflammation increases, causing an “early” stage of AS. Over time, the inflammation decreases, and bone tissue formation slowly starts. This is the intermediate stage of the AS. As the disease progresses, it causes ectopic chondroproliferation known as “syndesmophyte”. At the “later” stage of the disease, a bony bridge forms adjacent to the cortical bone surface called “ankylosis,” developing AS (45). (Created by Biorender.com).

Examination of individual vertebrae demonstrated that severe inflammation was never observed in conjunction with severe excessive tissue formation within the same joint, indicating that these disease features progressed sequentially rather than parallel. Nevertheless, the averaging of histology values indicates a convergence among the disease stages. Consequently, the model proposed by Tseng et al. forecasts that inflammatory lesions are expected to decrease in the later stages of the disease, characterized by the formation of syndesmophytes unless resolved (45) (**Figure 1**). This hypothesis is supported by magnetic resonance imaging (MRI) studies in AS patients, which indicate that (1) new syndesmophytes are more commonly found in vertebral corners where inflammation has subsided (51), and (2) 68% of syndesmophytes originated from vertebral corners are devoid of active inflammation during the two-year observation period (52). Nonetheless, the sensitivity thresholds of MRI for identifying mild inflammation must be acknowledged (53).

TNF may play a role in osteoproliferation and inflammation. Tseng et al. identified elevated TNF expression in the spines of PGISp mice during the stages of inflammation and excessive tissue development. The clinical heterogeneity within each vertebra may also be reflected in these higher levels, and local TNF levels may impact the regulation of osteoblasts at specific sites (45).

It has been demonstrated that hypertrophic chondrocytes can undergo direct differentiation into osteoblasts and osteocytes (54). The principal mechanism of excessive tissue growth in the PGISp model may be the direct transformation or ossification of chondrocytes, as exemplified by chondroidal ossification (43, 55). In the early stages, mature syndesmophytes are uncommon; however, they become more common as the disease progresses. This finding indicates that the transformation of cartilage to bone transpires gradually in PGISp mice, mirroring the prolonged duration between disease onset and radiographic alterations in AS patients (56). This indicates that, even with the inflammatory process being managed or decelerated, more osteoproliferation in that area will persist if inflammation is not addressed promptly to avert irreversible structural damage (45).

One recently identified mechanism that could be implicated in SpA is the tissue renin-angiotensin system (RAS). Angiotensin 1–7 may be a significant factor influencing the differentiation of bone cells. Atypical bone alterations in spondyloarthritis (SpA), angiotensin II receptor blockers (ARBs), and angiotensin-converting enzyme inhibitors (ACEis) exhibit distinct effects. RAS is necessary for osteoblast and osteoclast differentiation. Additionally, targeting RAS may be used to treat SpA (57).

## 3 Experimental mouse models for AS research

Radiography and MRI analysis have been used in several insightful investigations to characterize disease development (51, 58), although these modalities can only reveal noticeable structural abnormalities. Tissue samples from affected areas are essential for clarifying the cellular and molecular changes that influence the progression of the disease. Nonetheless, the availability of valuable clinical samples is restricted because of the difficulties associated with acquiring biopsies from axial skeletal sites (44).

A few investigations have been conducted on zygapophyseal joints after spinal procedures and sacroiliac joint biopsies from patients undergoing hip replacements (43, 59–62). Such investigations, while instructive, are constrained by sample size, anatomical location, and the fact that most patients were in the latexstages of the disease, at which point the inflammation-osteoproliferative shift had already occurred. Thus, the only practical method to understand the processes underlying disease progression in the axial skeleton is to use disease-relevant animal models. A robust approach for biologically mapping the complete trajectory of AS involves conducting time-course studies on spinal samples from animal models, particularly to examine the molecular interplay between inflammation and osteoproliferation (45).

No mouse model has been demonstrated to be appropriate for investigating the progression from inflammation to ankylosis in the axial skeleton, despite several animal models displaying characteristics similar to those observed in human diseases. Ankylosis was exclusively noted in transgenic rats exhibiting elevated β2-microglobulin expression, which was associated with decreased gastrointestinal disease and attenuated unfolded protein response (63). Transgenic rats that overexpress HLA-B27 and human β2-microglobulin exhibit spontaneous gastrointestinal illness and peripheral and axial inflammatory arthritis (47).

Two mice models that overexpress TNF, either through transgenic methods (hTNFtg) (64) or by enhancing TNF mRNA stability through the removal of the 3' ARE regulatory elements (TNFΔARE) (65), display systemic inflammation, gastrointestinal disorders, and sacroiliitis, yet do not independently develop ankylosis. C57BL/10 (66) and DBA/1 (67) mice are two murine models that spontaneously manifest ankylosing enthesopathy (ANKENT), albeit only in their peripheral joints. In SKG mice treated with curdlan or mannan, enthesitis, and osteoproliferation have also been observed (48). However, the correlations between these processes have not been clearly defined. In this model, entheseal proliferation and inflammatory infiltration were limited; glucocorticoid treatment (68) reduced inflammation, whereas etanercept treatment (69) did not affect ankylosis.

Notably, chondroproliferation and consequent activation of relevant bone morphogenetic protein signaling were exclusively prevented by early intervention, even though inhibiting TNF signaling prior to or after the onset of arthritis either cured or mitigated the condition (70). In the SKG mouse model, bone growth and erosion were shown to correlate with inflammation; however, the precise relationship between these factors over time remains unclear owing to the absence of comprehensive longitudinal research (48).

Due to the equivocal results from other animal models, Tseng et al.’s comprehensive investigation of the PGISp mice strongly indicates a direct path from inflammation to cartilage-bone development (45). This is the only inducible murine model that exhibits axial ankylosis and a significant immunological component. Injectable illness caused by PG extract from human cartilage mimics axial inflammation and ankylosis due to early inflammatory activation in symptoms and signs (46, 71). Inflammation of the entheses around the sacral and spinal joints is the first sign of axial illness in PGISp mice. The IVD erodes when inflammatory cells invade the intervertebral joint region, producing an invasive pannus. Mesenchymal cell growth and development of a collagen- and PG-rich matrix follow, with the latter having the potential to mineralize and cause ankylosis. These data demonstrate that the PGISp model is ideally suited for studying the etiology of AS because it is the typical clinical course of AS (72). Further characterization of axial disease pathology and the associated genetic abnormalities in the Wnt signaling system validate PGISp mice as a reliable model for bone formation due to AS-typical joint inflammation (44).

Pepelyayeva et al. (2018) found that mice deficient in the universal endoplasmic reticulum aminopeptidase 1 (ERAP1) gene eventually develop systemic osteoporosis, spinal cord inflammation, and spontaneous axial ankylosis (73). Additionally, similar to AS patients who show an increased vulnerability to inflammatory bowel disease (IBD) (74) and dysbiosis in the terminal ileum (75), ERAP1^−/−^ mice exhibited intestinal characteristics that appeared to make them more susceptible to chemically induced colitis and spontaneous gut dysbiosis. The aggregated findings suggest that the aforementioned mice models provide a significant and viable animal model for examining the pathogenesis of the predominant skeletal (40, 75–77) and intestinal symptoms seen in AS patients (75, 78). Potential therapeutic compounds targeting AS’s skeletal, immunological, and digestive symptoms of AS can also be tested in ERAP1^−/−^ mice. ERAP1^−/−^ mice represent a distinctive animal model for investigating interactions within the gut-bone-immune axis (73).

Recent research has demonstrated that bone pathology in aged CD4-Cre knockout (CD4-CKO) mice closely resembles that of AS. This is the first animal model of AS that can offer a significant platform for investigating new pathological bone formation in AS (79). The mechanistic delay in growth plate fusion was caused by the CD4-Cre-induced SHP2 deficit in proliferating chondrocytes, which subsequently differentiated into pre-hypertrophic and hypertrophic chondrocytes. A portion of the osteoproliferation and ectopic new bone formation was brought about by the active chondrogenesis that occurred in the growth plate and the enthesis that occurred through the BMP6/Smad1/5 signaling pathway. Joint stiffness and possibly lifelong impairment result from the formation of osteophytes bridging the joint cavity. In CD4-CKO mice, sonidegib, an SMO inhibitor, eliminated disorganized chondrogenesis and markedly reduced problematic new bone formation. The results indicate that focusing on chondrocytes in disorders is a possible way to stop the radiographic advancement of AS. This means that blocking chondrogenesis with sonidegib could be a functional medication-repurposing approach for treating AS (79).

Pathological osteogenesis was shown to occur as a result of the rapid osteogenic differentiation of mesenchymal stem cells (MSCs) from patients with AS (AS-MSC), and it was found that during this enhanced differentiation, AS-MSC induced TNF-α-mediated local inflammation (80, 81). The etiology of AS has been supported by observations demonstrating that high concentrations of TNF-α promote the initiation of increased directional migration of AS-derived mesenchymal stem cells (MSCs) in both *in vitro* and *in vivo* models (82). ELMO1 expression in AS-MSC was increased by TNF-α in a spondyloarthritis model, with higher expression *in vivo* in AS patients. Inhibiting ELMO1/DOCK Rac1 GEF activity with a small-molecule DOCK inhibitor called CPYPP demonstrated a remarkable anti-inflammatory therapeutic effects in SKG mice, which spontaneously develop chronic arthritis (82, 83).

## 4 Clinical biomarkers of AS

### 4.1 Extracellular metabolites

It is now common practice to measure inflammation using C-reactive protein (CRP), which is usually elevated in patients with AS compared to patients with nr-axSpA (1, 15, 84). An increase in CRP levels can be caused by a variety of pathogenic events, including the common cold or ongoing inflammation. Therefore, the localized pathogen-related inflammation present in diseased joints may not be detected by CRP. The intercellular spaces are filled with a network of molecules and fibrils called the extracellular matrix (ECM). During inflammation, the extracellular matrix (ECM) goes through significant changes and reflects pathogenic events, such as the influx of inflammatory cells, by reflecting these changes (85).

Local production of MMP-degraded ECM metabolites occurs during inflammation. ECM tissue turnover may be identified by serum biomarkers, which would represent regional pathogenic processes (86). Serum levels of MMP-3, MMP-8, and MMP-9 are elevated in patients with AS, particularly in those with higher disease activity and increased structural progression (87, 88).

Not surprisingly, nr-axSpA was more common than AS in several respects, including a higher frequency in females, a more rapid disease progression, less severe radiographic status, and reduced CRP levels (85). In contrast, the nr-axSpA subgroup had a higher prevalence of peripheral arthritis than the AS subgroup (13). Recently, de Winter et al. posited that 50 percent of patients with axSpA had axial and peripheral symptoms; nevertheless, no difference was observed between these patients and those with axial symptoms concerning radiographic sacroiliitis (89).

**Figure 2:**
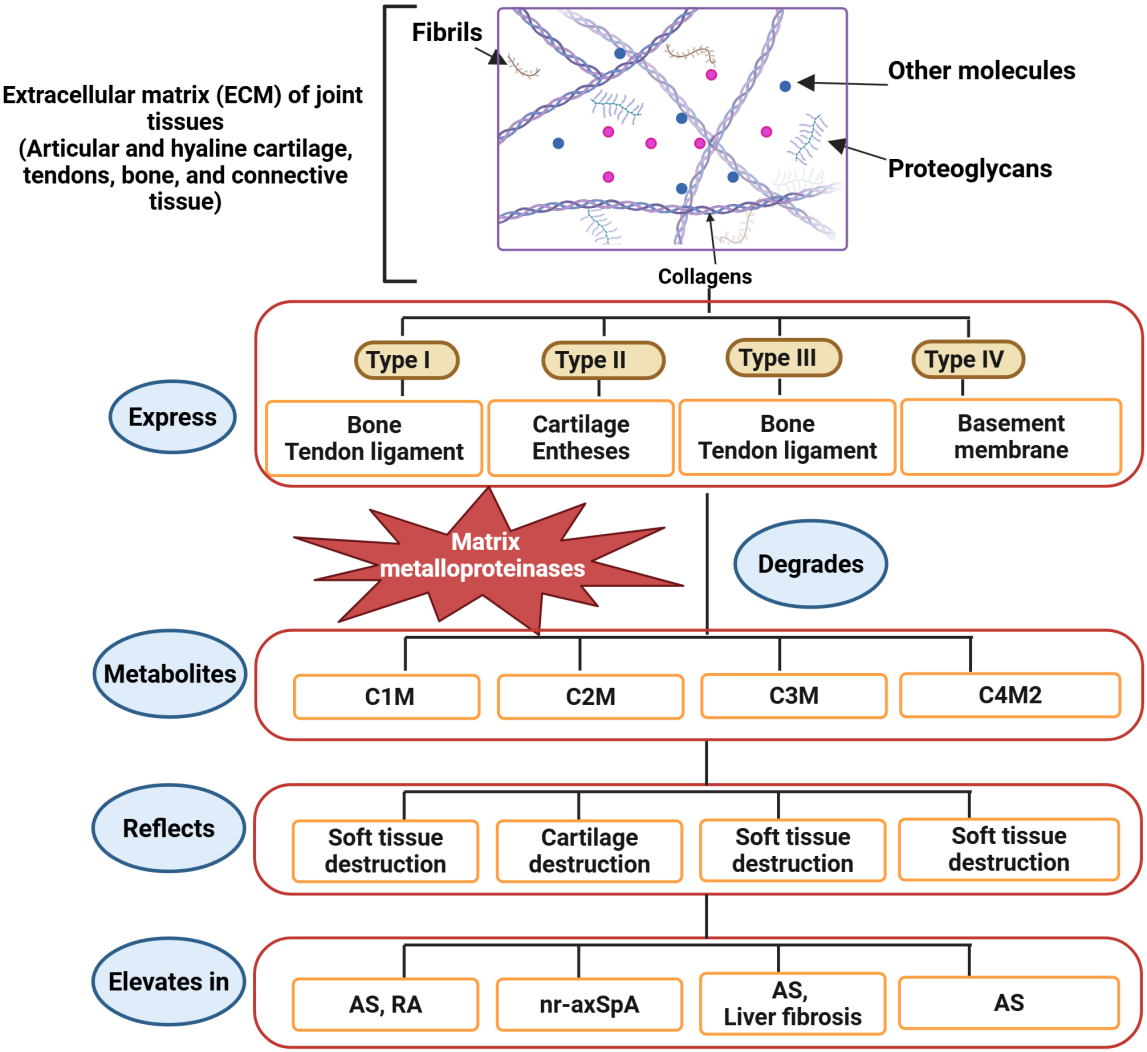
Recently identified biomarkers of axSpA and AS. The ECM of joint tissues comprises collagens, glycosaminoglycans, fibrils, and other molecules. Collagens are categorized into four types based on tissue expression. Bone and ligaments express type I and type III collagens. Type II and type IV collagens are expressed in cartilage, entheses, and basement membranes. MMPs, producing different metabolites, degrade the ECM. C1M, C3M, and C4M2 are metabolites of soft tissue destruction, whereas C2M is a metabolite of cartilage destruction. Therefore, the elevation of C1M, C3M, and C4M2 indicates axSpA, while the C2M increase indicates nr-axSpA, and all directly specify the development of AS (85). (Created by Biorender.com).

An increase in ECM turnover indicates the involvement of numerous processes in the immune-musculoskeletal pathophysiology of axSpA. Numerous joint tissues, such as articular and hyaline cartilage, bone, connective tissue, tendons, and type I, II, III, and IV collagens are expressed in the ECM. These collagens are susceptible to degradation by proteases. Bone, tendons, and ligaments contain collagen types I and III, whereas the basement membrane contains collagen type IV. The two primary tissues that produce collagen type II are the cartilage and entheses. Several metalloproteinases (MMPs) prominently produced during inflammation naturally degrade these collagens. Osteogenesis-inducing alterations and activation of other matrix metalloproteinases (MMPs) are facilitated by MMP-3, which is expressed locally in inflammatory tissues (90). Disease activity and progression are indicated by the serum levels of MMP-1, -2, -3, -8, and -9 (87, 88, 91) (**Figure 2**).

Immune cells, macrophages, and fibroblasts may be sources of MMPs. Hušáková et al. conducted a study wherein they discovered that the serum levels of ECM metabolites (C1M, C3M, and C4M2) exhibited a higher rate of ECM turnover in soft tissue and joint structures (85). All three indicators exhibited a robust connection with CRP and showed greater significance in AS patients than in nr-axSpA patients, perhaps indicating differing degrees of inflammation between the two disorders. In both nr-axSpA and AS patients, Hušáková et al. found elevated levels of C2M. Furthermore, neither the CRP levels nor the radiographic scores were correlated with the C2M biomarker, which was comparable in both groups (85). C1M is a metabolite connected to rapid structural advancement in RA (92), and C3M and C1M have recently been linked to disease activity in psoriatic arthritis (93). Decreased resistance to mechanical stress in an SpA model sustained entheseal inflammation, stimulating the production of new bone (94) (**Figure 2**).

Increased breakdown of type I, type II, and type IV collagens (C1M, C2M, and C4M2) may lead to inadequate development, contributing to biomechanical inadequacy in the surrounding structures, including joints and entheses. Even though AS patients had higher mean ECM metabolite levels than nr-axSpA patients, there was too much overlap (significant variation) in metabolite levels to effectively distinguish between the axSpA groups based solely on metabolite levels. The correlation between AS and nr-axSpA may be associated with disease activity, as individuals with elevated biomarker levels may show rapid progression, whereas those with diminished levels may demonstrate more stability (85).

A recent study found that in differentiating AS from nr-axSpA and asymptomatic controls, C3M performed better than C2M17. C3M also performed well in a study by Hušáková et al. When comparing asymptomatic controls to those with AS or nr-axSpA, CRP performed worse than C1M, C3M, and C4M2 (**Figure 2**). However, these metabolites should be included in a diagnostic panel for practical purposes to ensure adequate specificity and sensitivity in diagnosing and identifying disease subgroups. The findings of the research conducted by Hušáková et al. on C3M may suggest the existence of a novel serological biomarker that is capable of identifying both axSpA subtypes. Nonetheless, using C3M as a prospective diagnostic tool for AS and nr-axSpA requires further comprehensive research. Even though these results are promising, it is essential to determine if C3M is strong enough to predict the disease (85).

Patients with nr-axSpA did not have systemic cardiovascular abnormalities, such as atherosclerotic changes, unlike those with AS. Nevertheless, patients with nr-axSpA exhibiting extra-articular manifestations have elevated atherosclerotic plaques (95). In a sizable postmenopausal female epidemiologic cohort, higher levels of C1M were identified to be an independent mortality risk factor for several diseases, including cancer and cardiovascular diseases (95). According to the research conducted by Hušáková et al., the degradation of type I collagen was found to be more severe in women who had AS and nr-axSpA (85).

Men with AS are thought to experience cardiovascular issues more frequently than the general population (96), while both sexes are believed to have an equally elevated risk of cancer (97). A new meta-analysis indicates that patients with nr-axSpA and AS exhibited no differences in extra-articular symptoms (13), a finding supported by Hušáková et al. Notably, patients with a predominance of extra-articular manifestations exhibited higher C3M levels than those without extra-articular manifestations in nr-axSpA. This finding suggests that C3M may originate from the joint tissues and tissues involved in extra-articular manifestations. Both C1M and C3M appear to indicate changes in cardiovascular and fibro-proliferative processes (85).

The expression levels of matrix remodeling factors varied with the disease stage, indicating the deleterious impact on the early onset of disease. MMP3 (a stromelysin) and MMP13 (a collagenase) are two critical extracellular matrix remodeling enzymes that have been shown to be upregulated in animal models and patients with AS (88, 98, 99). Important bone matrix constituents, such as collagen 1 (Col1), bone sialoprotein, osteocalcin (OCN), and several other extracellular matrix-associated genes, were markedly upregulated in response to the strong matrix formation response (44). Further research should clarify the relationship between systemic involvement and axSpA-related collagen tissue turnover (85).

Released by articular chondrocytes, cartilage intermediate layer protein 1 (CILP-1) is deposited into the cartilage extracellular matrix. CILP-M is a neo-epitope of CILP-1 produced by matrix metalloproteinase (MMP). Proteolytic enzymes were used to break down human articular cartilage, resulting in the production of CILP-M levels. Recently, CILP-M, a novel neo-epitope biomarker, was created and validated for human blood samples (100). It measures a fragment of CILP-1 mediated by MMP-1, MMP-8, and MMP-12. CILP-M levels were increased in patients with RA and AS compared to healthy controls, and in patients with AS, these levels were reduced after TNF-α treatment. Based on these findings, CILP-M may be helpful in evaluating cartilage remodeling in degenerative joint disorders.

### 4.2 ERAP1 and immune cells

The discovery of the genetic link between AS and the HLA-B27 allele was reported about 40 years ago, conclusively linking the immune system to AS susceptibility (101). On the other hand, AS only develops in 1-5% of people with the HLA-B27 variant; therefore, there must be other risk factors (76). The HLA-B27 variant is present in more than 90% of patients with AS. The presence of ERAP1 polymorphisms and HLA-B27 show strong epistatic gene-gene interactions (35), even though HLA-B27 is considered to represent 23% of the genetic risk for AS (73).

As one of ERAP1’s primary functions is to trim peptides before they are loaded onto major histocompatibility complex (MHC-I) molecules, epistasis between ERAP1 and HLA alleles supports the theory that variations in antigen presentation pathways may contribute to AS pathogenesis (102, 103). Additionally, ERAP1 has been linked to the inhibition of innate and adaptive immune responses by Pepelyayeva et al. in 2018 (73) and others (104–106). The etiology of AS is also thought to involve gastrointestinal issues, which would increase the genetic and immunologic complexity of the condition (78).

ERAP1^−/−^ mice lack overall immune suppressive functions, as evidenced by their heightened innate and adaptive immune responses to different stimuli compared to WT mice (104–109). Although earlier studies showed that Foxp^3+^ Tregs are disturbed in AS (110, 111), Yuliya et al. failed to find variations in Foxp^3+^ Treg number or function in the peripheral tissues of ERAP1^−/−^ mice (73). Even though they did not completely eliminate the possibility that Foxp3+ Treg function could be altered by ERAP1 loss, their investigation unequivocally demonstrated that the “Tr1-like” cells, which are an essential subset of regulatory T cells, were significantly reduced in mice lacking ERAP1.

**Figure 3:**
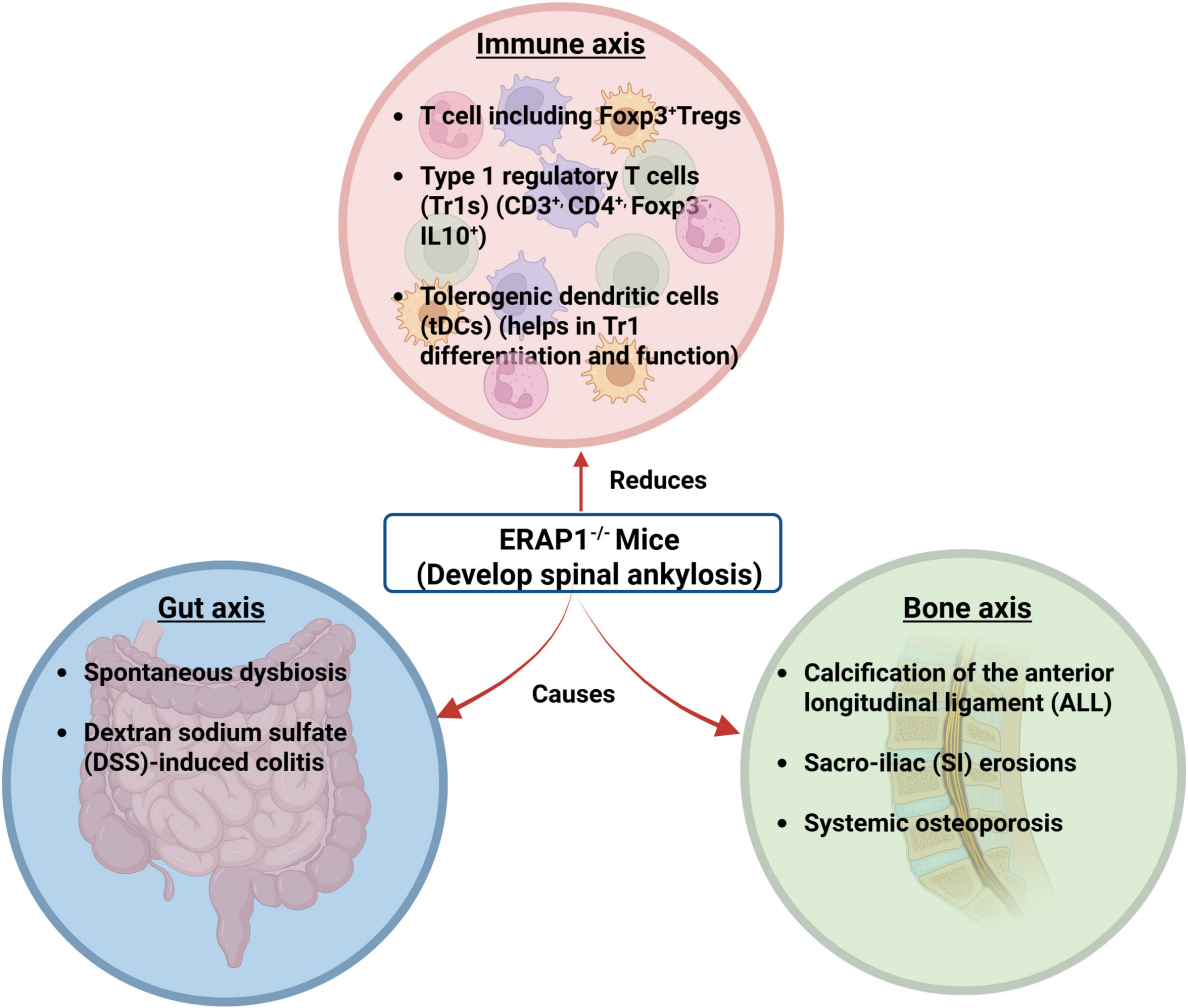
ERAP1^−/−^ mice showing interactions of the gut-bone-immune axis. ERAP1^−/−^ mice develop rapid and spontaneous onset of spinal ankylosis involving bone, gut, and immune axes. The bone axis causes calcification of the anterior longitudinal ligament (ALL), sacroiliac (SI) erosions, and systemic osteoporosis. The gut axis refers to spontaneous dysbiosis and dextran sodium sulfate (DSS)-induced colitis. In the immune axis, Foxp3+, CD3+, CD4+, Foxp3−, IL-10+, and tDCs are reduced (13). (Created by Biorender.com).

Tr1 cells are distinguished from other cells by their ability to suppress responses from peripheral T cells and antigen-presenting cells (APCs) and their capacity to release substantial amounts of IL-10 and TGF-β without expressing Foxp3 (112). Researchers also discovered that ERAP1^−/−^ mice had considerably fewer tolerogenic dendritic cells (tDCs) in their spleens. It is well established that tDCs stimulate the development of Tr1 cells among naive CD4+ T cells. With regard to the maturation of T cells and differentiation of Tr1 cells, it is believed that the expression of HLA-G on T cells and its interaction with ILT-2 and ILT-4 receptors on naive CD4+ T cells are both significant determinants (113, 114) (**Figure 3**).

Using whole-genome expression analysis, Haynes et al. aimed to understand the molecular alterations underpinning the progression from inflammation to bone formation (44). Changes were observed in matrix catabolic and anabolic pathways, as well as in inflammatory pathways. The levels of components of the TNF- and IL-1 pathways were both elevated. Genetic studies have linked both pathways to AS (103, 115). Psoriasis, which often coexists with AS in patients, has been linked to IL28ra (116). Both cell types (secreting interleukin-23 receptor-positive gamma/delta T cells) have been linked to SpA; Stat1 and Stat3 mediate TH1 and IL-17-associated signaling, respectively (117, 118) and are believed to be involved in the PGISp mice model (119, 120). There is a possibility that the elevated expression of these genes is related to the increased TH1 and IL-17-expressing cell activity that was observed in the experimental animals. Human AS has also been shown to be associated with STAT3 (121). These molecular patterns provide further evidence that the PGISp model mimics cellular alterations in AS, molecular patterns, and inflammation polarization (44).

### 4.3 Disc degeneration

ERAP1^−/−^ mice have been found to have heightened innate and adaptive immune responses to a variety of stimuli (104, 107, 108). Additionally, hematoxylin and eosin (H&E) staining revealed severe lumbosacral intracranial degeneration in ERAP1^−/−^ mice. This degeneration is characterized by local structure disruption and mononuclear cellular infiltrations in the nucleus pulposus (NP). A study using immunohistochemistry on the ERAP1^−/−^ spines revealed that the mononuclear infiltrates stained strongly for TNF-α and IL-23 in the cytoplasm (122). TNF-α is considered a significant factor in disc degeneration due to its ability to attract inflammatory cells to the IVD, destroy the extracellular matrix, and contribute to hyperalgesia and calcification associated with IVD disease (123) (**Figure 3**).

AS and diffuse idiopathic skeletal hyperostosis (DISH) were compared radiologically in a recent study employing whole-spine computed tomography (CT), including the spine and sacroiliac joint (SIJ) (124). Patients with AS often have complete SIJ fusion, whereas patients with DISH often have anterior/posterior bridging. However, a sizable proportion of patients with DISH have SIJ fusion. Individuals with AS are more prone to demonstrating smooth anterior spinal bridging in the lumbar region than those with diffuse idiopathic skeletal hyperostosis. Conversely, patients with DISH often have candle-wax-type bridging, which commonly occurs in the thoracic spine. Nevertheless, it is intriguing that a select number of patients with DISH exhibited smooth-type bridging, whereas a few patients with AS exhibited candle-wax-type bridging. Moreover, spinal facet fusion was present in a sizable proportion of patients with DISH. Therefore, these details should be considered when diagnosing AS or DISH (124).

### 4.4 Gut microbes

Patients with colitis have a threefold increased risk of developing clinical AS, and microscopic inflammatory damage to the gut mucosa is observed in up to 60% of patients with AS (125). Furthermore, it has been found that AS patients have different microbiota in the terminal ileum compared to healthy individuals (75). This finding implies that microbial interactions with the intestinal immune system could play a role in AS pathogenesis and extra-intestinal immunological disorders (126–128).

Cytokine production patterns have been shown to have a significant impact on gut immunity and AS etiology. Short-chain fatty acids (SCFAs) and ATP, which are metabolites generated from the gut microbiota, have been demonstrated in recent research to promote the growth and differentiation of T-helper 17 (Th17) cells and regulatory T cells (Tregs), respectively (129–131). Chronic inflammation is thought to be primarily caused by dysregulation of the interleukin-23 (IL-23)/Th17 signaling axis (132–135). This route, in short, is the polarization of naïve CD4+ T cells toward a Th17 phenotype, which is defined by a preponderance of IL-17 and IL-22 production. Transforming growth factor-beta (TGF-β), IL-23, and IL-6 facilitate this differentiation by triggering the IL-23/Th17 pathway (136). For instance, *Salmonella enteritidis* has been demonstrated to stimulate local Th17 responses in animal models of reactive arthritis (137), and *Chlamydia trachomatis*, a recognized cause of reactive arthritis, can promote IL-23 production (138). By showing that infiltrating monocytes constitute a significant contributor to elevated IL-23 expression in AS, Ciccia et al. identified IL-23 overexpression as a crucial aspect of the disease’s subclinical gut inflammation (132). It is suggested that the gut-joint inflammatory axis that underlies AS is significantly influenced by IL-23, IL-17, and IL-22 taken together (139).

Commensal bacteria are hypothesized to contribute to inflammatory illnesses such as RA, IBD, and multiple sclerosis (MS) and the immune system’s development and maintenance of skeletal and gut homeostasis (126–128, 140). Based on the findings of Yuliya et al.’s study on 16S rRNA fecal phenotyping, ERAP1^−/−^ mice naturally increased the diversity of bacteria in their feces, including several genera and phyla such as *Cyanobacteria, Actinobacteria, Prevotella, Odoribacter, Bacteroides, Lactobacillus plantarum YS2, Clostridiales*, and *Parabacteroides* (73).

In addition, *Lachnospiraceae, Christensenellaceae*, and *S24.7* genera were significantly underrepresented in ERAP1^−/−^ mice. Osteoporosis, similar to that in ERAP1^−/−^ mice (141), is caused by an overgrowth of Prevotella in HLA-B27 transgenic rats (142). Additionally, it has been noted that the terminal ileums of AS patients are enriched in *Prevotella* (75). Fecal samples of patients with AS have been found to contain an increased number of Bacteroides (143). The *Lachnospiraceae* and *Ruminococcaceae* families, as well as the *Actinomyces* and *Streptococcus* genera (75) and *Firmicutes phylum* (144), were found to be on the rise in the AS patient’s terminal ileum, but the same tendencies were not observed in ERAP1^−/−^ mice. Thus, in the future, detailed research will be required to broaden our comprehension of the effects of the human gut microbiota on AS because there is currently a dearth of information on this topic among individuals with AS. The connections between skeletal, immunological, and intestinal dysbiosis were best studied in ERAP1^−/−^ mice as a model system (73).

Recent research by Yulia et al. and others has shown that ERAP1 influences both the activity of antigen-specific T cells and the selection of immunodominant T cell epitopes (73, 107–109, 145). Due to decreased immunological tolerance that allowed abnormal microbial communities to populate the intestines of ERAP1^−/−^ mice, dysbiosis of the intestine in ERAP1^−/−^ mice may be potentially caused by ERAP1’s involvement in immunodominance. Cross-fostered ERAP1^−/−^ mice failed to minimize the severity of ankylosis or osteoporosis or to alleviate spinal inflammation, which minimizes the contribution of the gut microbiota to these phenotypes. However, it must be noted that because the levels of the genera *Christensenellaceae*, *Parabacteroides*, and *Bacteroides* were not adjusted in the cross-fostered ERAP1^−/−^ mice, these commensal microorganisms are likely responsible for the skeletal abnormalities that have been observed in these mice. Although there has been evidence of an association between communities of microbes (such as *Bacteroides fragilis* (146) and *Clostridium* species (128)) and the growth and function of Treg, their research also raises the possibility that Tr1 cells and dysbiosis might be related (**Figure 3**). However, more detailed studies are required to elucidate the link between Tr1 cells and dysbiosis (73).

### 4.5 Wnt signaling pathway and its inhibitors

The biological molecules causing these tissue changes are also of interest, especially from the perspective of potential therapeutics, as they reflect the gene expression changes found in the joint tissue. The Wingless (Wnt) signaling pathway has been shown to be a crucial regulatory pathway for bone-forming cells (osteoblasts) (147). The main critical antagonists of the Wnt pathway include sclerostin (SOST), secreted Fzd-related proteins, and Dickkopf-1 (DKK-1),which are unique to or substantially enriched in osteoblast-lineage cells.

**Figure 4:**
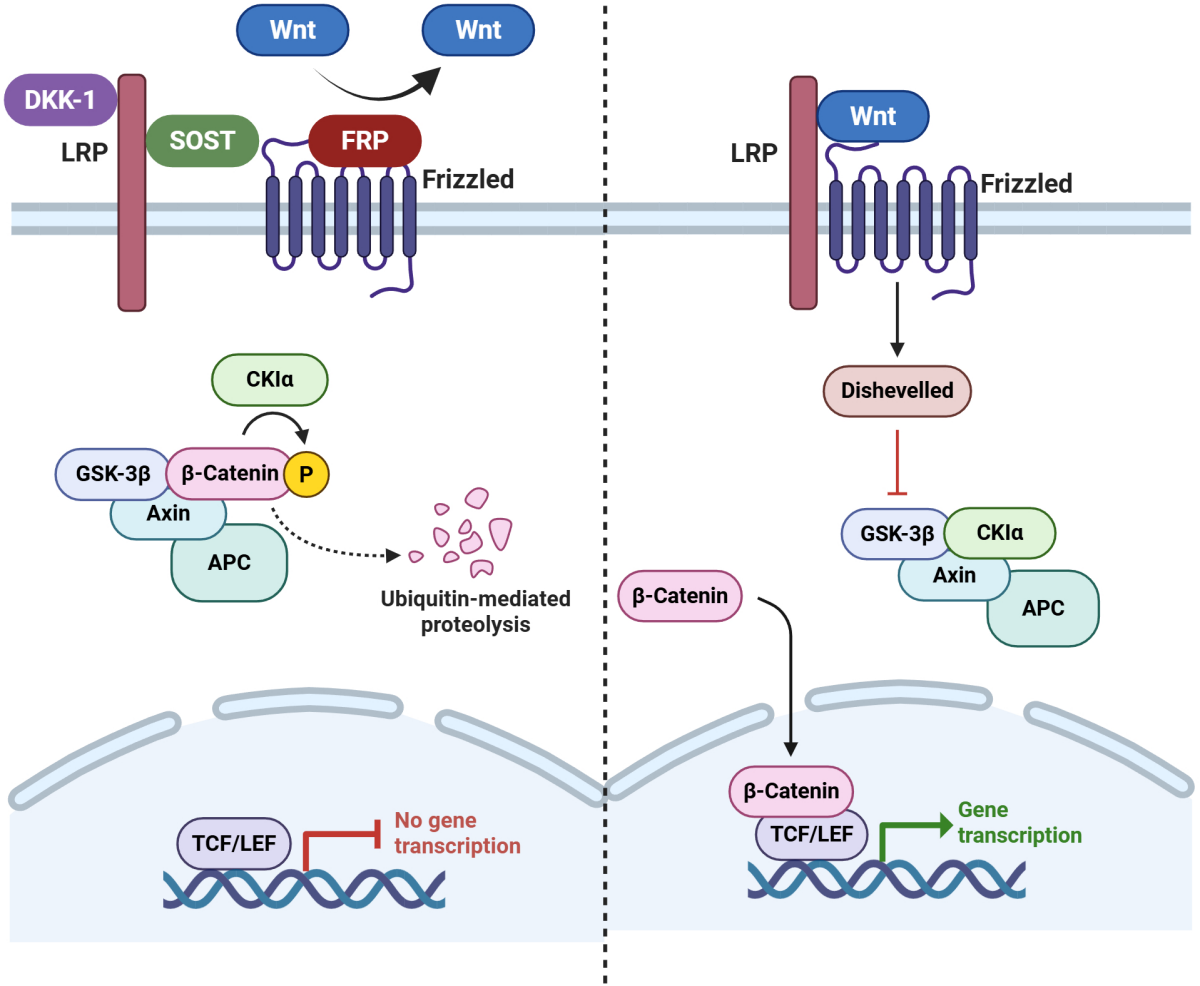
Wnt signaling pathway and its inhibitors. During canonical Wnt signaling in bone, soluble Wnts bind to their Frizzled (Fzd) receptors and LRP4/5/6 co-receptors in a ternary complex at the cell surface. This inhibits GSK-3 and allows β-catenin (β-cat) to accumulate. Upon entering the nucleus, an accumulation of β-cat is responsible for initiating the transcription of the gene of interest. When Wnt signaling is not active, GSK-3 predominantly phosphorylates the protein β-cat, which causes ubiquitination and proteasome-mediated destruction of the protein. Key Wnt pathway inhibitors in bone include secreted Fzd-related proteins, Dickkopf-1 (DKK-1), and sclerostin (SOST). These proteins bind to LRP4/5/6 to prevent them from interacting with the Wnt-Fzd complex or with Wnts to prevent them from participating in the process (180). (Created by Biorender.com).

Recent research has shown that DKK-1, a potent Wnt pathway inhibitor, is a crucial regulator in experimental models of joint injury and arthritis (148). Bone resorption has been linked to elevated DKK-1 levels, while new bone growth has been linked to decreased levels (149, 150). DKK-1’s function in pathological ossification was highlighted by the notable fusion of the sacroiliac (SI) joints that occurred when it was blocked in an animal model of arthritis (151). In contrast to individuals with rheumatoid arthritis (RA), who had high Dkk-1 levels, AS patients showed significantly lower Dkk-1 levels. A dynamic link between inflammatory signals and Wnt pathway regulation is also shown by the significant decrease in DKK-1 expression seen in RA patients after anti-TNFα treatment (148). Lower levels of DKK-1 (148) and SOST (152) have been observed in patients with AS. Ankylosis has also been shown to result from DKK-1 inhibition in a mouse model of spondylitis with high TNF expression (151) (**Figure 4**).

The first proof of enhanced Wnt signaling in a mouse model of SpA or AS was provided by Haynes et al. in 2012, who corroborated this hypothesis by exhibiting notably reduced amounts of DKK1 and SOST, two Wnt inhibitors (44). Recent studies, including both human patients and rodent models, have established a connection between the Wnt pathway, which is crucial for bone formation and homeostasis, and AS (45).

Numerous studies have documented that the Wnt/β-Catenin and TGF-β signaling pathways are crucial in the molecular pathophysiology of AS. The overexpression of programmed cell death 10 (PDCD10) in synovial cells revealed a transcriptome that may link this gene to these two signaling pathways (153). According to additional research, PDCD10 has been shown to control β-Catenin expression favorably. Synovial cell calcification can be facilitated by PDCD10, whose expression was found to be elevated in AS patients. There was a positive correlation between PDCD10 and the Bath Ankylosing Spondylitis Disease Activity Index (BASDAI) and the modified Stoke Ankylosing Spondylitis Spinal Score (mSASSS). As a biomarker for diagnosing and treating AS, PDCD10’s ROC analysis indicates that it may be utilized as a biomarker for diagnosing and treating AS (153).

### 4.6 Factors of bone metabolism

Receptor activator of nuclear factor kappa-B (RANK)/receptor activator of nuclear factor kappa-Β ligand (RANKL) and other bone metabolism-related substances, including SOST, Dickkopf-1, bone alkaline phosphatase (ALP), and bone morphologic protein, have been proposed as possible biomarkers for AS (154). An indicator of the overall impact of bone turnover is probably bone-specific ALP among these. Placental, intestinal, and germ cell ALP and tissue non-specific alkaline phosphatase (TNAP) are the four different isoenzymes that comprise the homodimeric enzymes known as ALPs, which are crucial for catalytic activity.

Concerning the function of ALP, TNAP was found to be linked to bone mineralization and may be a therapeutic target for developing new bone in AS patients. Additional evidence suggests that bone-specific TNAP may serve as a predictive biomarker for radiographic progression in patients with AS, as derived from the study’s finding of a significant correlation between the two variables across two cohorts and radiographic progression. The modified Stoke Ankylosing Spondylitis Spinal Score (mSASSS) measurement from five years and three months ago revealed the highest beta coefficient. In the linear mixed model, the serum ALP level was significantly correlated with the mSASSS at 5 years and 3 months before radiographic changes. Several lab experiments have confirmed that new bone growth in AS occurs without inflammation. Moreover, neither systemic nor local inflammation affected the expression of osteoprotegerin, RANKL or RANK in the peripheral synovitis of spondyloarthritis (154).

## 5 Current treatment strategies for AS

The lack of knowledge about the molecular pathways causing disease progression is a significant contributor to the inadequate therapeutic options for severe AS (45). Within the context of spondylitis and its radiographic progression, the benefits of early intervention are widely recognized and appreciated despite the availability of a variety of treatments. After 16 weeks of treatment with Infliximab (a monoclonal antibody against TNFα), 61% of patients with a short duration of disease (13.4 months) showed a 40% improvement from baseline. Only 47% of patients with an extended disease duration (7.7 years) achieved the same outcome after 24 weeks of treatment (155). A higher rate of radiographic progression was associated with delaying TNFα intervention therapy when compared to individuals who started treatment within 10 years after the disease started (156). Non-steroidal anti-inflammatory drug (NSAID) therapies and early intervention were both beneficial in the treatment of AS. Following prolonged high-dose NSAID therapy, the radiographic progression of AS patients with a short disease duration of five years was found to be lower (157) compared to those with a long disease duration of eleven and a half years (158). Early AS/spondyloarthritis patients achieve better clinical outcomes with anti-inflammatory therapy than individuals with more advanced disease (45).

With a mean retention duration of 59 months in axSpA, a recent study found that TNF inhibitor golimumab (GOL) had a significantly higher retention rate when compared with adalimumab (ADA), etanercept (ETN), and certolizumab-pegol (CZP). For this case, things like being male, not having any peripheral diseases, and starting the treatment early were linked to better SC-TNFi retention in axSpA (159). Anti-TNF medication has a high success rate in reducing inflammation, but ankylosis may still progress (160–163). According to recent studies, long-term TNF inhibition may help halt the progression of AS (156, 158, 162–166). However, to date, the radiographic progression of AS has not been stopped or reversed by any treatment.

Interleukin-17 (IL-17) is a pro-inflammatory cytokine that is associated with matrix damage, tissue inflammation, and autoimmune. As a result, IL-17 became an essential therapeutic target for immune system disorders. Most of the recent decade’s research has concentrated on IL17A inhibitors. These small molecules have the potential to improve clinical symptoms in AS patients drastically. Furthermore, no notable adverse effects or infections have been reported in patients after consuming IL-17A inhibitors, and they can be used as a complementary and alternative treatment to TNF inhibitors. As a result, IL-17 has become the primary focus of AS research (167).

While IL-23 blockers were beneficial for psoriasis, they have been found ineffective even for patients with AS (168, 169). A monoclonal antibody generated in humans called utekinumab specifically targets the p40 component of IL-12 and IL-23. However, AS clinical studies were abruptly halted since they could not show any improvement in symptoms (170). While an IL-23 blocker did not affect inflammation, it did successfully prevent the onset of disease symptoms in rats (171). Furthermore, there are other factors affecting IL-17A production besides the IL-23/IL-17A axis. As a result, inhibiting IL-23 might not be sufficient to stop the inflammation that is caused by IL-17 (170). Clinical trials were conducted on the anti-IL12/23 inhibitors, IL-6 receptor inhibitors, IL-1 receptor antagonists, T cell co-stimulation inhibitors, phosphodiesterase-4 inhibitors, and anti-CD20 antibodies; however, none of these drugs demonstrated adequate efficacy in treating axial AS disorder (172).

JAK inhibitors have been approved by the US Food and Drug Administration for the treatment of AS. In a phase 3 trial, upadacitinib, a selective JAK1 inhibitor, administered at a dose of 15 mg once daily, demonstrated good response in AS refractory to a prior history of biologic disease-modifying antirheumatic medications, with ASAS 40 at 45% at 14 weeks (173).

Cytokine, granulocyte-macrophage colony-stimulating factor (GM-CSF), improved RA signs and symptoms in a phase II trial and may cause joint injury by increasing osteoclasts and matrix metalloproteinases (174). Namilumab, a monoclonal antibody that targets GM-CSF, is presently being evaluated in a phase IIa study, with the primary endpoint of ASAS 20 at week 12 (170).

SOST selectively inhibits Wnt signaling in skeletal tissue by being expressed in osteocytes and chondrocytes (179). As observed in animal models and human disease, inactivating mutations in SOST enhance Wnt signaling and increase bone mass and strength (180). SOST is a promising therapeutic target in bone disease due to its tissue-specific expression (**Figure 4**). The only abnormalities manifest in individuals with SOST-inactivating mutations are directly related to excessive bone mass (179). In patients with osteoporosis, antibody treatments targeting SOST have shown increased bone density and decreased bone turnover markers (181, 182). However, daily administration of human recombinant (rSOST) had no impact on the axial skeleton or peripheral joint disease development or severity in PGISp mice, according to a study by Haynes et al. in 2015 (39).

The study conducted by Haynes et al. found that rSOST was ineffective at inhibiting excess tissue formation. This could be due to several factors, including its low stability, inability to reach joints, or inadequate dosage (39). A transgenic mouse for human SOST, in which a BAC encoding the human SOST gene was inserted, is osteopenic (179). Human SOST has previously been shown to be active in a mouse system. In this model, overexpressing human SOST significantly blocked Wnt signaling, reducing osteoblast activity and bone formation. It’s probable that extra bloodstream proteins bonded to rSOST and prevented it from functioning at the joint (39).

SOST is still a promising treatment option for AS, even though it failed to significantly impact bone growth when injected in the mice in the above-discussed study. A number of studies, both on mouse models and on human patients, strongly suggest that the regulation of SOST is an essential component in the process of bone growth in AS. Serum and biopsies collected from individuals with AS significantly decrease SOST expression (152, 183). The expression of SOST has significantly diminished in serum and biopsies obtained from individuals with AS (184). Spondylitis develops in mice treated with DKK1-neutralizing antibodies, albeit this also lowers SOST levels (185).

Furthermore, PGISp mice exhibit downregulated SOST expression in the intervertebral joints (44). SOST might require further stabilization or bone-specific targeting to be more effective as a treatment. The specific expression of SOST in osteoblasts and osteocytes, rather than inherent bone-targeting abilities, is responsible for SOST’s ability to target bone in non-pathological situations. SOST can be complexed with a drug that targets bone, like bisphosphonate, to deliver SOST effectively to bone. A mouse model of metastatic breast cancer has previously shown success with this strategy of coupling a treatment to a bisphosphonate for targeting bone (186).

Recombinant proteins have been used in very few reports of *in vivo* investigations that focus on the skeleton. The 2.5 g dosage is comparable to that of earlier research. Therefore, an inadequate dosage may have contributed to the ineffectiveness of rSOST in changing the progression of the disease in untreated mice. Future research should examine the effects of greater dosages, considering safety and toxicity profiles. Using exogenous rSOST in other mice models may provide insights into the role and importance of Wnt signaling in pathological bone formation and its potential therapeutic implications (39). **Table 1** summarizes all the available treatment strategies for AS.

**Table 1:** Current therapeutics available or under trial for AS.

Treatment strategies	Examples	Mode of action	Efficacy	References
TNF inhibitors	Infliximab, adalimumab, etanercept, golimumab, certolizumab pegol	Small molecules that inhibit TNF, reducing pro-inflammatory signaling and modulating interferon pathways.	Rapid symptom relief, improved function, and enhanced quality of life.	(167, 168, 175)
IL-17 inhibitors	Secukinumab, ixekizumab, netakimab, bimekizumab brodalumab	Monoclonal antibodies targeting IL-17 cytokines to reduce inflammation.	Improves symptoms, spinal mobility, and quality of life; complementary or alternative to TNF inhibitors.	(167, 168, 170, 175)
IL-12/23 blockers	Ustekinumab, Risankizumab, guselkumab	Monoclonal antibodies targeting p40 subunit of IL-12 and IL-23 to suppress inflammation	Clinical trials in AS were halted due to a lack of symptom improvement, as they prevented disease onset in rats without affecting inflammation.	(167, 170, 174)
JAK inhibitors	Tofacitinib, upadacitinib, filgotinib	Small molecules block JAK1 signaling, reducing inflammatory protein production.	Reduces inflammation and improves quality of life.	(170)
GM-CSF inhibitors	Namilumab	Monoclonal antibody targeting GM-CSF, a pro-inflammatory agent.	Improved RA symptoms in phase II trials; under study for AS.	(170)
T cell co-stimulation modulator	Abatacept	Blocks effector T-cell responses, reducing inflammation.	Clinical trials showed insufficient efficacy in axial disease.	(176)
IL-6 receptor inhibitors	Sarilumab, tocilizumab	Inhibit IL-6 receptors, reducing acute phase response and inflammation.	Studied clinically but lacked sufficient efficacy in axial disease.	(174)
IL-1 receptor antagonists	Anakinra	Prevent prostaglandin E2 formation and bone resorption stimulation.	Clinical trials showed insufficient efficacy in axial disease.	(177)
Anti-CD20 antibody	Rituximab	Induces direct cell death or cytotoxicity via complement or antibody-dependent cellular cytotoxicity.	Clinical studies showed insufficient efficacy in axial disease.	(174)
PDE-4 inhibitor	Apremilast	Suppresses inflammatory responses by inhibiting phosphodiesterase-4.	Clinically evaluated but did not show sufficient efficacy in axial disease	(178)
α4β7 integrin antagonists	Vedolizumab	Blocks α4β7 integrin to reduce inflammation.	Effective in reducing inflammation	(168)
COX-2 inhibitors	Celecoxib	Inhibits cyclooxygenase-2 enzyme, reducing eicosanoid production and pain.	Provides pain relief with fewer gastrointestinal side effects	(175)
DMARDs	Methotrexate, sulfasalazine	Inhibit immune responses of monocytes, T cells, and B cells.	Provides symptom relief and slows disease progression.	(175)
NSAIDs	Ibuprofen, naproxen	Inhibit cyclooxygenase-2 enzymes, reducing pain and inflammation.	Effectively reduce pain and inflammation.	(175)
Human rSOST	–	Selectively inhibits Wnt signaling, reducing bone mass.	No impact on axial skeleton or peripheral joint disease	(39)

N.B, TNF inhibitors, tumor necrosis factor inhibitors; IL-17 inhibitors, interleukin 17 inhibitors; IL-12/23 inhibitors, interleukin 12/23 inhibitors; JAK inhibitors, janus kinase inhibitors; GM-CSF, granulocyte-macrophage colony stimulating factor; IL-6 receptor inhibitors, interleukin-6 inhibitors; IL-1 receptor antagonist, interleukin-1 antagonist; Anti-CD20 antibody, anti- cluster of differentiate 20 antibody; PDE-4, phosphodiesterase-4; COX-2 inhibitors, cyclooxygenase-2 inhibitors; DMARDs, disease-modifying anti-rheumatic drugs; NSAIDs, non-steroidal anti-inflammatory drugs; Human rSOST, human recombinant sclerostin

## 6 Conclusion

The intricate interplay between inflammation and tissue remodeling in AS highlights the complexities of its pathogenesis, especially as shown in the PGISp mice model. In contrast to the better-known endochondral and intramembranous ossification processes, this model has shown that chondroidal ossification is the primary driver of excessive tissue formation. According to recent research, the inflammatory environment in AS may have a dual role in the degeneration of IVDs and the promotion of abnormal ossification pathways that result in the formation of syndesmophytes and, eventually, ankylosis. The ERAP1^−/−^ mice model significantly advances our understanding of AS by closely mimicking the disease’s intestinal, immune, and skeletal manifestations. This model offers a significant framework for exploring the fundamental AS processes and evaluating prospective treatment approaches.

In contrast to controls, individuals with radiographically confirmed axSpA variations had elevated blood levels of ECM metabolites such as C1M, C2M, C3M, and C4M2, indicating that these biomarkers may signify disease activity. The significant connection between blood levels of MMP-degraded collagen products and clinical disease activity shows the complex interplay between tissue turnover and inflammatory processes in AS. In particular, C3M is a promising biomarker for differentiating AS from non-radiographic axSpA (nr-axSpA), necessitating additional research into its clinical applications.

The dysregulation of Wnt signaling pathways in AS models is a compelling target for therapeutic intervention. Dysregulation of Wnt signaling in a mouse model of AS increases tissue proliferation, contributing to syndesmophyte formation and ankylosis. This imbalance is believed to have a role in osteoproliferation, which supports the therapeutic targeting of Wnt signaling in AS. The biological activity of rSOST treatment, which appears to modulate SOST levels in the joints, paralleling the downregulation of SOST observed in human AS patients, highlights the potential of therapeutic agents targeting Wnt signaling. Moreover, the downregulation of SOST observed in the PGISp mouse model closely resembles the downregulation of SOST observed in humans, as previously described. Nonetheless, more dosage-response investigations are needed to clarify the mechanisms by which SOST and associated pathways might be adeptly modified to influence tissue development in AS (39).

In conclusion, the findings from these models offer a compelling rationale for early intervention with effective anti-inflammatory therapies to mitigate damage caused by inflammation and reduce the incidence of osteoproliferative events and joint fusion in an individual with AS. As these insights will be essential for developing targeted therapeutic strategies, future research should concentrate on identifying essential proteins and molecular pathways that regulate the transition from inflammation to bone formation. Eventually, learning more about the molecular basis of AS will help the development of personalized medicine methods, which will lead to better outcomes for people who have this debilitating disorder.
